# High physical activity and high sedentary behavior increased the risk of gestational diabetes mellitus among women with excessive gestational weight gain: a prospective study

**DOI:** 10.1186/s12884-020-03299-8

**Published:** 2020-10-07

**Authors:** Heng Yaw Yong, Zalilah Mohd Shariff, Barakatun Nisak Mohd Yusof, Zulida Rejali, Jacques Bindels, Yvonne Yee Siang Tee, Eline M. van der Beek

**Affiliations:** 1 Department of Nutrition and Dietetics, Faculty of Medicine and Health Sciences, Universiti Putra Malaysia, 43400 Seri Kembangan, Selangor Malaysia; 2 Department of Obstetrics and Gynaecology, Faculty of Medicine and Health Sciences, Universiti Putra Malaysia, 43400 Seri Kembangan, Selangor Malaysia; 3 Danone Nutricia Research, Uppsalalaan 12, 3584 CT Utrecht, The Netherlands; 4 Danone Specialized Nutrition (Malaysia) Sdn. Bhd, Suites 8.01 & 9.01, Levels 8 & 9, The Garden South Tower, Mid Valley City, Lingkaran Syed Putra, 59200 Kuala Lumpur, Malaysia; 5 Department of Pediatrics, University Medical Centre Groningen, University of Groningen, Groningen, The Netherlands

**Keywords:** Physical activity, Trajectory, Sedentary behaviour, Gestational diabetes mellitus

## Abstract

**Background:**

Although physical activity (PA) in pregnancy benefits most women, not much is known about pregnancy-related changes in PA and its association with gestational diabetes mellitus (GDM) risk. The aim of this study was to identify the trajectory of PA during pregnancy and possible associations with the risk of GDM.

**Methods:**

This was a prospective cohort study of 452 pregnant women recruited from 3 health clinics in a southern state of Peninsular Malaysia. PA levels at the first, second, and third trimester were assessed using the Pregnancy Physical Activity Questionnaire. GDM was diagnosed at 24–28 weeks of gestation following the Ministry of Health Malaysia criteria. Group-based trajectory modeling was used to identify PA trajectories. Three multivariate logistic models were used to estimate the odds of trajectory group membership and GDM.

**Results:**

Two distinct PA trajectories were identified: low PA levels in all intensity of PA and sedentary behavior (Group 1: 61.1%, *n* = 276) and high PA levels in all intensity of PA as well as sedentary behavior (Group 2: 38.9%, *n* = 176). Moderate and high intensity PA decreased over the course of pregnancy in both groups. Women in group 2 had significantly higher risk of GDM in two of the estimated logistic models. In all models, significant associations between PA trajectories and GDM were only observed among women with excessive gestational weight gain in the second trimester.

**Conclusions:**

Women with high sedentary behavior were significantly at higher risk of GDM despite high PA levels by intensity and this association was significant only among women with excessive GWG in the second trimester. Participation in high sedentary behavior may outweigh the benefit of engaging in high PA to mitigate the risk of GDM.

## Background

Healthy women with uncomplicated pregnancies are encouraged to engage in regular physical activity (PA) before, during, and after pregnancy, although modification to exercise routines may be necessary due to the anatomic and physiologic changes that occur during pregnancy [1]. The current PA recommendations for pregnant women are based on the evidence and recommendation for healthy adults, in which healthy pregnant women should begin or continue at least 150 min of moderate-intensity aerobic activity per week during pregnancy (i.e., equivalent to brisk walking) [2]. Vigorous-intensity exercise is not recommended for previously inactive women or women who engage in only moderate-intensity exercise, while women who are currently engaged in vigorous activity may continue with this level of activity during most of their pregnancy.

Despite the recommendation for pregnant women to be active, both retrospective and prospective studies showed that most pregnant women (> 50%) do not meet the recommended PA [3, 4] and physical activities consistently decrease during pregnancy with the most extensive changes occurring during the third trimester [5]. In the UK and US, only 3–15% of pregnant women met the recommended PA compared to 24–26% of non-pregnant women of childbearing age [6–8]. There is considerable evidence that PA during pregnancy has beneficial effects for both the mother and fetus [9–12]. For the mother, PA may help prevent excessive gestational weight gain (GWG), gestational diabetes mellitus (GDM), pre-eclampsia, cesarean deliveries, and improve mental health [9, 10]. Regular PA may also help to maintain cardiovascular fitness during pregnancy and positively impact postpartum recovery [13, 14]. Previous studies have shown that physical exercise during pregnancy promotes improvements in the cardiovascular adaptation of the fetus (i.e., decreased fetal heart rate and increased fetal heart rate variability) [15–19], as well as increase fetoplacental growth rate and further promote healthier birth weight [11].

To date, only a few longitudinal studies evaluated PA in cohort setting, and these studies suggested a variation in timing and magnitude of the decline in PA during pregnancy [20, 21]. The benefits of PA and the disadvantages of physical inactivity among the general population are well recognized [22–24]. However, evidence on the effect of PA during pregnancy on GDM risk is inconsistent [25–30]. While several studies showed an inverse association [25, 27, 30], others did not find any association [26, 28, 29]. However, studies reporting an inverse association, have been limited by their cross-sectional examination of PA and GDM [25–27], making it difficult to ascertain the cause-effect relationship. In addition, most of the prior studies focused on the association between total PA level or individual domain of PA (i.e., household/caregiving and occupational activity) with GDM risk. None of these studies examined intensity group-based PA trajectories over the course of pregnancy, which describe the combinations of several intensities of PA (i.e., PA level and sedentary behavior). Thus, the aim of this study was to identify PA patterns trajectories from before pregnancy to during pregnancy and the risk for GDM of the PA trajectory groups.

## Methods

### Study design and location

**Fig. 1:**
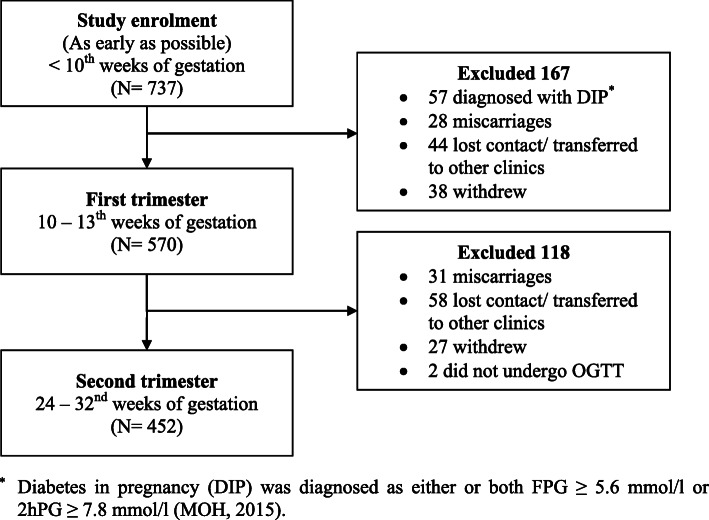
Recruitment of study respondents. ^*^ Diabetes in pregnancy (DIP) was diagnosed as either or both FPG ≥ 5.6 mmol/l or 2hPG ≥ 7.8 mmol/l (MOH, 2015)

SECOST (Seremban Cohort Study) was a prospective study in which pregnant women were followed-up through 1 year postpartum, and their infants were followed-up every 6 months until 2 years old. Women in the first trimester (10 – 13th weeks of gestation) of pregnancy were recruited from three maternal and child health (MCH) clinics in Seremban District, Negeri Sembilan, Malaysia. Detailed descriptions of the study methodology have been previously published, and only a brief overview is provided here. All pregnant women were eligible to participate unless they had one or more exclusion criteria [31]. Of the 737 women enrolled in the study, 452 (61.3%) women completed follow-up until the oral glucose tolerance test (OGTT) was performed. Two hundred and 85 women were excluded because they were diagnosed with diabetes in pregnancy (DIP) (*n* = 57), had a miscarriage or stillbirth (*n* = 59), withdrew due to health/personal reasons (*n* = 65), moved to other clinics or loss of contact (*n* = 102), and 2 women did not undergo OGTT. The final sample comprised of 452 pregnant women (Fig. 1). Ethical approval was obtained by the appropriate local ethics committees. All participants provided written informed consent prior to data collection.

### Measurements

#### Physical activity

PA at each time point (pre-pregnancy, first, second, and third trimester) was assessed using a modified version of the Pregnancy PA Questionnaire (PPAQ) [32]. The PPAQ consisted of items on the frequency and intensity of PA and time spent engaged in 35 activities in four domains: household/caregiving, occupational, sports/exercise, and transportation. Activity intensities were determined based on the Compendium of Physical Activities [33]; activities identified as having a different intensity during pregnancy were assigned a modified intensity value [34]. The average daily energy expenditure for each activity was calculated by multiplying the amount of time spent in each activity with an established metabolic equivalent (MET) score for each activity (MET-hours per day). Activities were then categorized according to intensity (light, moderate, and vigorous) and sedentary behavior. The MET-hours per day of all intensity PA activities and sedentary behavior were used to determine the group-based PA trajectory.

#### Gestational Diabetes Mellitus (GDM)

A standard two-point diagnostic 75 g OGTT was performed at 28 – 32nd weeks of gestation. A 2-ml fasting venous blood was drawn by a clinic staff nurse before ingestion of a standard glucose solution to obtain fasting plasma glucose (FPG). Another 2 ml of venous blood was drawn at 2-h after the ingestion of standard glucose solution. All blood samples were sent for analysis on the same day to determine FPG and 2-h plasma glucose (2hPG) concentration. GDM was diagnosed if FPG was ≥5.6 mmol/l or/and 2hPG was ≥7.8 mmol/l according to the Ministry of Health (MOH) Malaysia guideline [35].

#### Other variables

Socio-demographic information included age, education level, ethnicity, employment, and monthly household income. Obstetrical information (e.g., gravidity, parity, medical history GDM, and family history of diabetes mellitus) was obtained from medical records. Height was measured at study enrolment, while weight was measured at each study visit using a standard instrument (SECA digital weighing scale and SECA body meter) and standard procedures. Women were requested to recall pre-pregnancy body weight. Pre-pregnancy body mass index (BMI) (kg/m^2^) was calculated as pre-pregnancy weight divided by the square of height and classified according to the recommendation of the World Health Organization: underweight (< 18.50 kg/m^2^), normal weight (18.50–24.99 kg/m^2^), overweight (25.00–29.99 kg/m^2^) and obese (≥ 30.00 kg/m^2^) [36]. The rate of GWG in the first and second trimester was defined as the average weekly weight gain in that trimester and then classified according to the 2009 US Institute of Medicine (IOM) guidelines, as inadequate, adequate, and excessive [37].

### Statistical analysis

PA trajectories were analyzed using group-based multi-trajectory modeling performed with a STATA plugin by using CNORM distribution for continuous data [38]. Both linear and quadratic trajectories for group 1 and group 2 were tested. Three different models (2, 3, and 4 trajectory groups) were tested for linear, quadratic and cubic specifications for trajectory shape until the best fitting model was established. The final number of trajectory groups was designated based on the average of Bayesian information criteria (BICs), and the proportion of estimated trajectory groups (the smallest group includes at least 5% of patients), as model fit statistics [39, 40]. Average posterior probability of 0.70 for the within-group membership was used to indicate internal reliability [40]. Trajectory analysis gives each participant a probability of belonging to each defined PA trajectory group. Based on these probabilities, the participants were assigned to the trajectory group where they had the highest probability of belonging to a particular group. All groups showed sufficiently high average posterior probability of individuals belonging to each of the groups (0.80–0.85). Two trajectory groups were finally identified and labelled as group 1: “low PA levels in all intensity of PA and sedentary behavior” (61.1%) and group 2: “high PA level by intensity, as well as high sedentary behavior” (38.9%).

Chi-square test of independence or Fisher’s exact test and Independent t-test were used to assess the association between women characteristics (socio-demographic, obstetrical, anthropometric measurements, and energy intake) with PA trajectory groups and GDM risk, respectively for continuous and categorical variables. All variables (education level, employment, household income, parity, pre-pregnancy BMI and rate of GWG in the second trimester) which were significant in univariate analysis were adjusted in multivariate analysis. The analyses of the association between GDM risk and PA trajectory groups was performed using binary logistic regression analyses to obtain odds ratios (ORs) and 95% confidence intervals (CIs). The persistently low PA in all intensity trajectory group (group 1) was set as the reference category in the outcome variable. Adjusted models were constructed as below: Model 1 adjusted for the only gestational week at the time of blood sampling. Model 2 adjusted for model 1 with education level, employment, and household income. Model 3 adjusted for covariates of model 2 in addition to biological factors, such as parity, pre-pregnancy BMI and rate of GWG in the second trimester. To investigate if PA trajectory groups differed across education level, employment, household income, parity, pre-pregnancy BMI and rate of GWG, separate interactions were tested by adding product terms to the model. Only the rate of GWG in the second trimester showed a significant interaction effect between PA trajectory and GDM risk. Further stratified analyses were performed for any significant interaction term in the association between PA trajectory and GDM risk. In sensitivity analyses, the association between PA trajectory and GDM risk was investigated among women in group 2. Statistical analysis was performed using STATA® 13. The statistical significance level was set to *p* < 0.05.

## Results

**Fig. 2:**
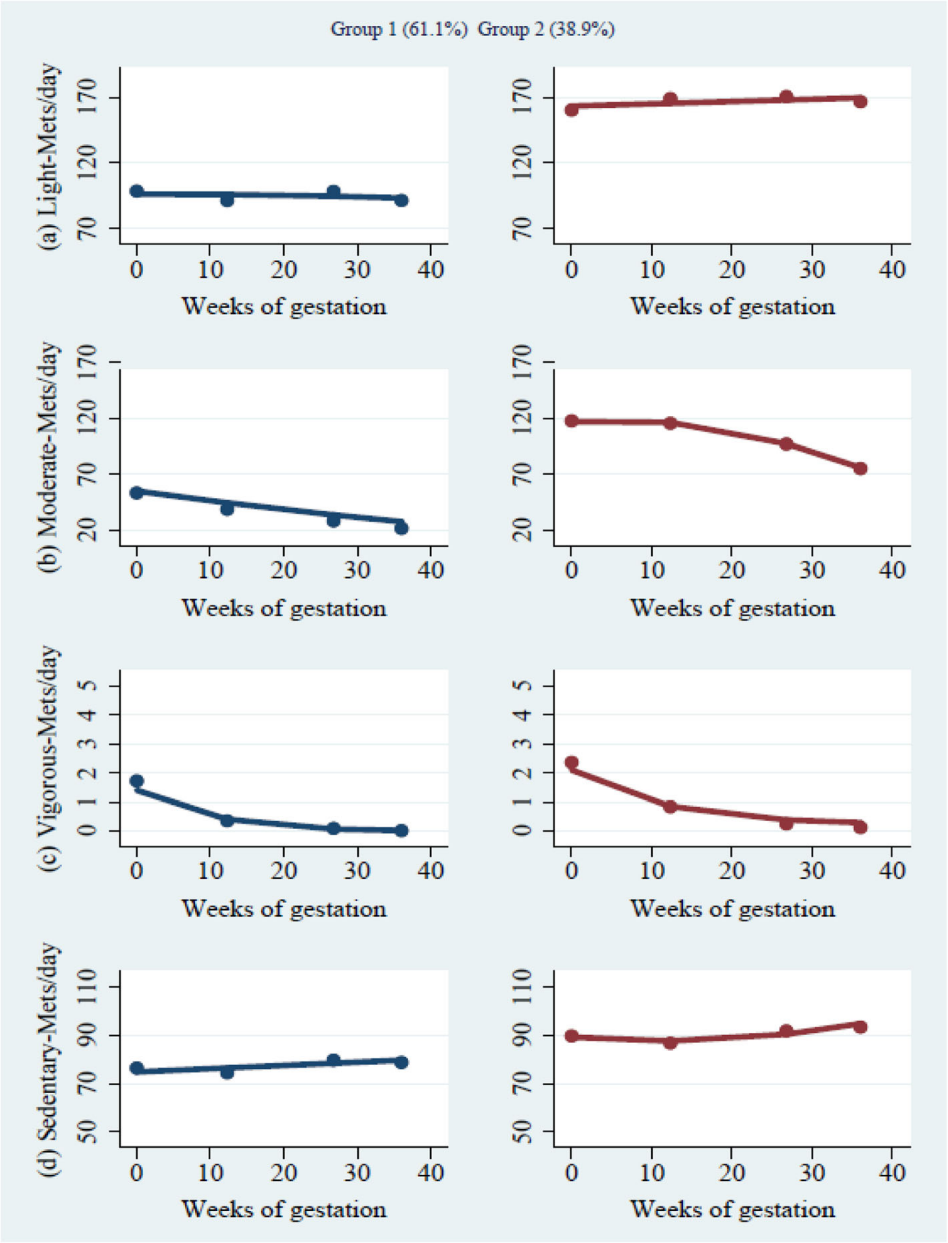
Mutli-trajectory model (physical activity trajectories by intensity. Note. **a** Moderate intensity (3.0 – < 6.0 METs); **b** Light intensity (1.5–3.0 METs); **c** Vigorous (≥ 6.0 METs); Sedentary (< 1.5 METs). Group 1 – Low PA levels in all intensity of PA and sedentary behavior (*n* = 276). Group 2 – High PA levels in all intensity of PA and sedentary behavior (*n* = 176)

Figure 2 shows the PA trajectory of women before and during pregnancy. Two trajectory groups were identified. Group 1, comprised of 61.1% of the sample, is labeled as “low PA levels in all intensity of PA, as well as sedentary behavior” due to low involvement in all intensity physical activities and overall a lower levels of sedentary behavior that increases slightly over the course of pregnancy. Group 2 (38.9% of the sample), highest on all intensity physical activity, as well as sedentary behavior, is named as “high PA level by intensity, as well as higher levels of sedentary behavior” also increasing over the course of pregnancy. Supplementary Table 1 shows the differences in the duration of physical activity between group 1 and group 2.

Table 1 presents the characteristics of the women by PA trajectory. Women in group 2 had significantly higher proportion with tertiary education and above (26.7%), were more likely to be employed (79.5%), and had a middle range of household income (42.0%) as well as GDM cases (14.8%) than women in group 1 (tertiary education and above = 18.1%; employed = 62.3%; middle household income = 27.9%; GDM cases = 8.0%). Women in group 2 had significantly higher mean gravidity (2.67 ± 1.44), and parity (1.44 ± 0.09) than women in group 1 (gravidity = 2.34 ± 1.47; parity = 1.09 ± 0.08). There was no significant difference in age, ethnicity, history of GDM, family history of DM, height, pre-pregnancy BMI, rate of GWG in the second trimester and enegy intake in the first and second trimester between group 1 and group 2. Overall, 28.1–39.6% of women achieved the minimum 150 min of weekly moderate intensity activity as recommended for pregnant women [2].

**Table 1:** Characteristics of women by trajectory groups (*n* = 452)

Characteristic	Trajectory Group		***p***-value
	Group 1 (***n*** = 276, 61.1%)	Group 2 (***n*** = 176, 38.9%)	
Age at study entry (years)	29.91 ± 4.62	30.45 ± 4.39	0.21
≤ 30	161 (58.3)	93 (52.8)	0.48
31–35	75 (27.2)	56 (31.8)	
> 35	40 (14.5)	27 (15.4)	
Ethnicity			
Malay	31 (11.2)	20 (11.4)	0.96
Non-Malay	245 (88.8)	156 (88.6)	
Education level			
Secondary and lower	140 (50.7)	67 (38.1)	0.02^*^
STPM/ Matric/ Diploma/ Certificate	86 (31.2)	62 (35.2)	
Tertiary and above	50 (18.1)	47 (26.7)	
Employment			
Housewife	104 (37.7)	36 (20.5)	0.001^**^
Working	172 (62.3)	149 (79.5)	
Household income (RM)^a^			
Low (< 3860)	191 (69.2)	96 (54.5)	0.01^¶**^
Middle (3860–8319)	77 (27.9)	74 (42.0)	
High (≥ 8320)	8 (2.9)	6 (3.4)	
**Obstetrical information**			
Gravidity	2.34 ± 1.47	2.67 ± 1.44	0.01^*^
1	108 (39.1)	34 (19.3)	0.001^**^
2	62 (22.5)	67 (38.1)	
≥ 3	106 (38.4)	75 (42.6)	
Parity	1.09 ± 0.08	1.44 ± 0.09	0.02^*^
0	123 (44.6)	40 (22.7)	0.001^**^
1–2	116 (42.0)	104 (59.1)	
≥ 3	37 (13.4)	32 (18.2)	
History of GDM			
No	262 (94.9)	160 (90.9)	0.09
Yes	14 (5.1)	16 (9.1)	
Family history of DM			
No	217 (78.6)	127 (72.2)	0.12
Yes	59 (21.4)	49 (27.8)	
**Anthropometric measurements**			
Height (m)	1.57 ± 0.06	1.56 ± 0.05	0.10
Pre-pregnancy weight (kg)	58.22 ± 12.32	58.63 ± 14.14	0.75
Pre-pregnancy BMI (kg/m^2^)	23.76 ± 4.81	24.01 ± 5.12	0.60
Underweight (< 18.5)	29 (10.5)	19 (10.8)	0.66
Normal (18.5–24.9)	152 (55.1)	93 (52.8)	
Overweight (25.0–29.9)	65 (23.6)	38 (21.6)	
Obese (≥ 30.0)	30 (10.8)	26 (14.8)	
Rate of gestational weight gain (GWG) (kg/week)			
Second trimester	0.39 ± 0.20	0.41 ± 0.26	0.34
Inadequate	89 (32.2)	54 (30.7)	0.82
Adequate	93 (33.7)	57 (32.4)	
Excessive	94 (34.1)	65 (36.9)	
Total GWG (kg)	11.53 ± 6.14	11.35 ± 5.61	0.75
Inadequate	104 (38.0)	64 (36.4)	0.64
Adequate	106 (38.7)	64 (36.4)	
Excessive	64 (23.3)	48 (27.2)	
Energy intake			
First trimester (kcal/day)	1572 ± 531.91	1583 ± 579.12	0.84
Second trimester (kcal/day)	1977 ± 687.55	1919 ± 647.29	0.16
**Maternal glucose level**			
Oral glucose tolerance test (OGTT)			
Gestational weeks at OGTT performed	28.02 ± 0.15	28.01 ± 0.33	0.66
Fasting plasma glucose (FPG) (mmol/L)	4.35 ± 0.57	4.41 ± 0.48	0.55
2-h plasma glucose (2hPG) (mmol/L)	5.92 ± 1.50	5.98 ± 1.53	0.67
GDM according to MOH criteria^b^	22 (8.0)	26 (14.8)	0.02^*^

Note. ^a^ 1 USD = RM 4.18 ^b^ GDM according to MOH criteria, either of both FPG ≥ 5.6 mmol/l or 2hPG ≥ 7.8 mmol/L

^*^p < 0.05

^**^p < 0.001

Table 2 presents the associations between PA trajectories and GDM risk. Women in group 2 were at a significant higher risk to develop GDM with adjusted OR of 1.98–2.01. However, the association was not significant after adjusting for parity, pre-pregnancy BMI, and rate of GWG in the second trimester. In addition, there was an interaction effect between the rate of GWG in the second trimester and PA level on GDM risk. Further stratified analyses showed that the significant association between women in group 2 had higher risk of GDM was only found among women with excessive rate of GWG in the second trimester (aOR = 2.37, 95% CI = 1.02–5.54) (Table 3). Sensitivity analyses were run among women in group 2, and the result remains similar (Supplementary Table 2). Interestingly, women in group 2 had a lower rate of GWG in the first trimester (0.18 ± 0.42 kg/week), but a higher rate of GWG in the second trimester (0.41 ± 0.26 kg/week), compared to group 1 (rate of GWG first trimester = 0.21 ± 0.37 kg/week; rate of GWG second trimester = 0.39 ± 0.20 kg/week). About 36.9% of women in group 2 had excessive rate of GWG in the second trimester (data not shown).

**Table 2:** Adjusted odds ratios and 95% confidence intervals for GDM risk among physical activity trajectory groups (*N* = 452)

Physical activity trajectory groups	GDM					
	Model 1		Model 2		Model 3	
	Adjusted OR [95% CI]	***p***-value	Adjusted OR [95% CI]	***p***-value	Adjusted OR [95% CI]	***p***-value
Group 1	1.00		1.00		1.00	
Group 2	2.01 [1.10–3.66]	0.02^*^	1.98 [1.11–3.60]	0.02^*^	1.78 [0.92–3.41]	0.06
**Interaction term**^a^						
PA trajectory x rate of GWG at second trimester	2.27 [1.63–6.21]	0.01^*^	2.13 [1.59–5.69]	0.01^*^	2.08 [1.45–5.47]	0.01^*^

Note. Non-GDM as reference

Model 1: Adjusted for gestational week at the time of blood sampling

Model 2: Adjusted for covariate in model 1 + education level, employment, and household income

Model 3: Adjusted for covariates in model 2 + parity + pre-pregnancy BMI + rate of GWG in the second trimester

^a^Only significant interaction terms are reported

^*^p < 0.05

**Table 3:** Adjusted odds ratios and 95% confidence intervals for GDM risk among physical activity trajectory groups stratified by rate of GWG in the second trimester

	GDM					
	Model 1		Model 2		Model 3	
	Adjusted OR [95% CI]	***p***-value	Adjusted OR [95% CI]	***p***-value	Adjusted OR [95% CI]	***p***-value
**Inadequate rate of GWG in the second trimester (*n*** **= 143)**						
Group 1	1.00		1.00		1.00	
Group 2	1.08 [0.30–3.87]	0.91	1.07 [0.28–4.03]	0.93	0.95 [0.24–3.77]	0.95
**Adequate rate of GWG in the second trimester (*n*** **= 150)**						
Group 1	1.00		1.00		1.00	
Group 2	2.32 [0.61–19.33]	0.16	2.29 [0.45–17.23]	0.27	2.19 [0.32–14.91]	0.42
**Excessive rate of GWG in the second trimester (*n*** **= 159)**						
Group 1	1.00		1.00		1.00	
Group 2	2.42 [1.09–5.40]	0.03^*^	2.45 [1.08–5.58]	0.03^*^	2.37 [1.02–5.54]	0.04^*^

Note. Non-GDM as reference

Model 1: Adjusted for gestational week at the time of blood sampling

Model 2: Adjusted for covariate in model 1 + education level, employment, and household income

Model 3: Adjusted for covariates in model 2 + parity + pre-pregnancy BMI

^*^p < 0.05

## Discussion

In the present study, women in group 2 were at significantly higher risk for GDM compared to women in group 1. Despite the higher PA levels in all intensity of PA (light to vigorous), the women in this group also showed higher sedentary behavior than group 1. Most women in group 2 as compared to group 1 were employed (79.5% vs. 55.4%), with about half (50.1% vs. 26.1%) having an office-based occupation (e.g., managerial/professional, administrative or clerical work) that involves desk work or sitting. Similarly, previous studies also showed that women who engaged in high levels of sedentary activities (e.g., TV watching, sitting at work or in vehicles, internet surfing, reading) were at increased risk for maternal hyperglycemia [20, 41, 42]. High levels of sitting may also occur alongside unhealthy behaviors, such as consuming high energy snack foods, which could lead to increased total energy intake and subsequently metabolic disorders [43–45], or more frequent snacking/eating that increase the metabolic challenge even if overall no increase in total energy intake. The quality of carbohydrates in meals and snacks might also be an important factor for glucose metabolism [46], however, this study did not measure the quality of carbohydrate intake. Although the association between sedentary behavior and GDM risk is not completely understood, sedentary behavior may affect maternal glycemia through directly altering glucose metabolism at the cellular level [47] that favors an insulin-resistant state [48]. Thus, increased PA during pregnancy may be associated with reduced GDM risk, while sedentary lifestyle, even if combined with increased activity may be associated with increased risk of GDM. Future investigations should focus on the variation in occupational sitting across different jobs during pregnancy, as well as the effects of occupational sitting on pregnancy outcomes.

The present study found that women showing high sedentary behavior were at significantly higher risk of GDM despite high PA levels (group 2). This finding highlights that participation in high sedentary behavior may outweigh the benefit of engaging in high PA in relation to the risk of GDM. Similarly, a recent study by Dieberger et al. (2020) [49] also found that overweight and obese pregnant women with more sedentary time had higher fasting glucose, insulin level, insulin sensitivity and insulin secretion than women with less sedentary time and this association could be due to the glucose-insulin axis effect [49]. This finding further supports the current guidelines for PA during pregnancy [1, 50] that recommends women to be physically active but more importantly to also limit sedentary behaviors. Future pregnancy guidelines could consider more specific recommendations on sedentary behavior, such as duration or time of sedentary behavior. As the observed sedentary behavior among women in this group was occupational sitting, suggesting the need for reducing sitting time in the office environment by including activity-permissive work practices (e.g., standing desk).

The present study reported an interaction effect of rate of GWG in the second trimester on the association between PA level trajectories on GDM risk. Specifically, women in group 2 with excessive rate of GWG in the second trimester showed a higher risk to develop GDM. It is also worthwhile to note that women in group 2 had a lower rate of GWG in the first trimester, in contrast to the higher rate of GWG in the second trimester compared to group 1. About 36.9% had excessive rate of GWG in the second trimester. Yong et al. (2017) [51] showed that gaining weight at a rate within the recommended range, but not dramatically gaining weight at only one of the trimesters (either the second or the third trimester) is important in order to achieve optimal maternal and child health [51]. Although the benefits of PA during pregnancy are well known, this study showed that being physically active might not be the most determining factor of GDM risk. Pregnant women should engage in a healthy lifestyle that includes healthy eating, being physically active, limiting sedentary activities but perhaps more importantly gaining appropriate gestational weight to reduce possible health risks during pregnancy.

Pregnancy is a period where most women might have a low PA level and reduce their PA levels over time [52]. In the present study, about two-thirds (61.1%) of the women had low PA in all intensity levels (Group 1). Previous studies also reported that only a small percentage of women were actively engaged in sports/exercise or recreational activities during pregnancy [53–55]. The relatively low levels of sports/exercise during pregnancy are likely to be associated with cultural norms for appropriate activity behaviors, particularly during late pregnancy. Women in both groups dramatically decreased their vigorous-intensity PA already in early pregnancy. The reduction in PA may be due to interpersonal health-related reasons, such as tiredness, shortness of breath, musculoskeletal problems, or physical restraints experienced by women as the pregnancy progresses [56, 57]. Also, an estimated 39.1% of women in this group were experiencing the first pregnancy. Thus, it is also possible that these women had relatively lower sports/exercise levels due to concerns related to pregnancy complications, such as premature labor or harming the baby from being active.

Group 2 was named as “high PA level by intensity, as well as high sedentary behavior”. Women in this trajectory had significantly higher gravidity or parity compared to women in group 1. About 42.6% of women in this group had three or more pregnancies. Thus, it is plausible that the higher PA level observed was due to these women having knowledge of PA from experiences. Knowledge of lifestyle behavior from previous pregnancy experiences will help in achieving an optimal pregnancy outcome in the next pregnancy [58]. It is also possible that the sedentary behavior among women in this group was worksite-induced sedentariness as about 79.5, and 26.7% of women in this group were employed and tertiary education and above. This finding is in line with previous studies whereby tertiary employees most likely to spend their daily time seated in front of computers [59, 60]. Thus, there is an urgent need to create a suitable culture of regular PA, but more importantly to reduce sedentary time at the workplace.

Limitations of this study should be noted. There was a tendency for self-report bias, as the PA level was self-reported. Although some misclassification of PA was possible, misclassification would be nondifferential and would be expected to bias the risk estimate toward the null because of the prospective design of this study. This study did not specifically assess the types of physical activity such as aerobic, muscle or bone strenghthening and stretching, which could possibly explain the association between PA trajectory and GDM risk. However, it is unlikely that women in this study would participate in strenuous activities such as weight training or endurance sport during pregnancy. A standardized diagnosis criteria of GDM is important to produce data that are comparable across study populations. The diagnostic criteria of GDM used in this study was based on the 2013 Perinatal Care guideline of the Ministry of Healthy Malaysia. Thus, there is a possibility that the GDM data were subjected to misclassification error. As several potential lifestyle-related variables (e.g. eating habits, quality of carbohydrate) were not included, this study cannot completely rule out the possibility of residual confounding. Despite these limitations, the present study clarified the related modifiable factors of participating in PA during pregnancy among pregnant Malaysian women. This finding might apply to the lifestyle of similar Asian pregnant women.

## Conclusions

Two trajectories of PA during pregnancy were identified in this study, of which one-third (group 2) had persistently higher levels of PA in all intensity as well as higher sedentary behavior, and these women were also significantly at greater the risk of GDM. Furthermore, this study also found that the significant association between high levels of PA and GDM risk was only observed among women with excessive GWG in the second trimester. These findings highlight the important role of sedentary behavior, whereby high sedentary behavior may outweigh the benefits of engaging in high PA in relation to the risk of GDM. Cumulatively, these findings suggest that women with excessive GWG could benefit more from reduction of sedentary behavior than stimulation of PA. Further investigation is required to understand the effects of the combination of high PA levels and high sedentary activities on GDM risk, but strategies to tackle low PA during pregnancy may be required. Besides, the study findings also have implications for the development of PA and sedentary guidelines for pregnant women.

## Supplementary information

**Additional file 1: Supplementary Table 1.** Duration of physical activity level by trajectory groups (*n* = 452). **Supplementary Table 2.** Adjusted odds ratios and 95% confidence intervals for GDM risk stratified by rate of GWG in the second trimester among women in group 2 (*n* = 176).

## Data Availability

The datasets generated and/or analysed in the current study are not publicly available due to ethical restrictions related to protecting patient confidentiality, but are available from the corresponding author on reasonable request.

## Abbreviations

PA: Physical Activity
GDM: Gestational Diabetes Mellitus
GWG: Gestational Weight Gain
SECOST: Seremban Cohort Study
MCH: Maternal and Child Health
OGTT: Oral Glucose Tolerance Test
DIP: Diabetes In Pregnancy
MOH: Ministry of Health
MREC: Medical Research Ethics Committee
PPAQ: Pregnancy Physical Activity Questionnaire
MET: Metabolic Equivalent
FPG: Fasting Plasma Glucose
2hPG: 2-h plasma glucose
BMI: Body Mass Index
IOM: Institute of Medicine
OR: Odds Ratios
CI: Confidence Intervals
