# Supplementary material for: High physical activity and high sedentary behavior increased the risk of gestational diabetes mellitus among women with excessive gestational weight gain: a prospective study
Source: BMC Pregnancy Childbirth. 2020 Oct 7;20:597. doi: 10.1186/s12884-020-03299-8 (PMC7541260; doi:10.1186/s12884-020-03299-8)
Supplement: Supplementary file 1 — Additional file 1: Supplementary Table 1. Duration of physical activity level by trajectory groups (n = 452). Supplementary Table 2. Adjusted odds ratios and 95% confidence intervals for GDM risk stratified by rate of GWG in the second trimester among women in group 2 (n = 176). [file 12884_2020_3299_MOESM1_ESM.docx]

|  | **Trajectory Groups^¥^** | | **p-value** |
| --- | --- | --- | --- |
|  | **Group 1**  **(n= 276, 61.1%)** | **Group 2**  **(n= 176, 38.9%)** |  |
|  | **mean ± SE (min/d)** | |  |
| **By intensity** |  |  |  |
| Sedentary (< 1.5 METs) |  |  |  |
| Pre-pregnancy | 480.78 ± 15.40 | 506.34 ± 17.82 | 0.38 |
| First trimester | 423.99 ± 14.18 | 489.57 ± 17.27 | 0.01* |
| Second trimester | 446.87 ± 14.29 | 505.14 ± 17.10 | 0.01* |
| Third trimester | 443.16 ± 14.11 | 525.80 ± 18.27 | 0.001** |
| Light (1.5–3.0 METs) |  |  |  |
| Pre-pregnancy | 349.98 ± 13.12 | 581.16 ± 22.92 | 0.001** |
| First trimester | 329.37 ± 12.93 | 632.89 ± 25.09 | 0.001** |
| Second trimester | 335.85 ± 11.07 | 617.20 ± 20.39 | 0.001** |
| Third trimester | 307.87 ± 9.77 | 576.46 ± 21.18 | 0.001** |
| Moderate (3.0–< 6.0 METs) |  |  |  |
| Pre-pregnancy | 142.59 ± 8.78 | 295.60 ± 16.93 | 0.001** |
| First trimester | 103.21 ± 7.36 | 276.83 ± 16.87 | 0.001** |
| Second trimester | 89.16 ± 7.27 | 248.27 ± 16.98 | 0.001** |
| Third trimester | 76.59 ± 7.25 | 189.92 ± 14.55 | 0.001** |
| Vigorous (≥ 6.0 METs) |  |  |  |
| Pre-pregnancy | 13.22 ± 2.59 | 17.02 ± 3.39 | 0.37 |
| First trimester | 5.28 ± 0.90 | 10.76 ± 2.47 | 0.05 |
| Second trimester | 4.59 ± 0.54 | 6.59 ± 0.66 | 0.37 |
| Third trimester | 2.28 ± 0.87 | 5.73 ± 0.35 | 0.01* |
| **By type** |  |  |  |
| Household/ caregiving |  |  |  |
| Pre-pregnancy | 272.74 ± 12.24 | 503.57 ± 24.40 | 0.001** |
| First trimester | 254.52 ± 11.06 | 526.05 ± 25.22 | 0.001** |
| Second trimester | 243.47 ± 10.43 | 523.86 ± 20.62 | 0.001** |
| Third trimester | 223.30 ± 8.25 | 481.60 ± 18.26 | 0.001** |
| Occupational |  |  |  |
| Pre-pregnancy | 260.61 ± 14.15 | 378.28 ± 16.43 | 0.001** |
| First trimester | 236.84 ± 14.01 | 374.64 ± 19.46 | 0.001** |
| Second trimester | 257.31 ± 14.52 | 351.82 ± 17.01 | 0.001** |
| Third trimester | 236.39 ± 14.36 | 322.34 ± 17.10 | 0.001** |
| Sports/ exercise |  |  |  |
| Pre-pregnancy | 7.14 ± 0.91 | 10.22 ± 1.32 | 0.05 |
| First trimester | 4.33 ± 0.67 | 8.82 ± 1.22 | 0.01* |
| Second trimester | 6.20 ± 0.87 | 10.02 ± 1.17 | 0.05 |
| Third trimester | 7.65 ± 1.07 | 9.19 ± 1.22 | 0.75 |

**Supplementary Table 1: Duration of physical activity level by trajectory groups (n=452)**

*p<0.05, **p<0.001

**Supplementary Table 2: Adjusted odds ratios and 95% confidence intervals for GDM risk stratified by rate of GWG in the second trimester among women in group 2 (n =176)**

|  | **GDM** | | | | | |
| --- | --- | --- | --- | --- | --- | --- |
|  | **Model 1** | | **Model 2** | | **Model 3** | |
|  | **Adjusted OR [95% CI]** | **p-value** | **Adjusted OR [95% CI]** | **p-value** | **Adjusted OR [95% CI]** | **p-value** |
| Rate of GWG in the second trimester |  |  |  |  |  |  |
| Inadequate vs. Normal | 1.15 [0.27 – 4.87] | 0.85 | 1.24 [0.28 – 5.45] | 0.78 | 1.05 [0.22 – 5.06] | 0.95 |
| Excessive vs. Normal | 4.68 [1.48 – 13.93] | 0.01^*^ | 4.75 [1.46 – 11.47] | 0.01^*^ | 4.11 [1.23 – 10.82] | 0.02^*^ |

Note. Non-GDM as reference.

Model 1: Adjusted for gestational week at the time of blood sampling

Model 2: Adjusted for covariate in model 1 + education level, employment, and household income

Model 3: Adjusted for covariates in model 2 + parity + pre-pregnancy BMI

*p< 0.05
